# Distribution, Sources, and Ecological Risk Assessment of Microplastics in the Lower Minjiang River

**DOI:** 10.3390/toxics13121033

**Published:** 2025-11-29

**Authors:** Liqin Bao, Jiayi Hao, Wenbin Pan

**Affiliations:** College of Environment & Safety Engineering, Fuzhou University, Fuzhou 350116, China; 230627099@fzu.edu.cn (L.B.); haojiayi0317@163.com (J.H.)

**Keywords:** lower Minjiang River, microplastics, distribution characteristics, sources, ecological risks

## Abstract

Microplastics, as a pervasive emerging pollutant, pose a critical threat to freshwater ecosystems and have emerged as a pressing global environmental concern. This study employed methods such as microscopic observation and Raman spectroscopy analysis to characterize the abundance, morphology, and polymer composition of microplastics in surface water and sediments from the lower Minjiang River (Fujian Province, China) in July and November 2024. By integrating socioeconomic indicators with water quality parameters, we dissected the pollution sources, and employed the Pollution Load Index (PLI), Polymer Chemical Toxicity Hazard Index (PHI), and Potential Ecological Risk Index (PERI) to quantify ecological risks in the study area. Findings reveal that the lower Minjiang River exhibits moderate microplastic contamination compared to domestic and international river systems, with mean abundances of 19.90 ± 1.56 n/L (flood-season surface water), 22.87 ± 1.32 n/L (dry-season surface water), and 728.17 ± 20.51 n/kg (dry-season sediments). Spatiotemporal dynamics demonstrate significantly higher microplastic loads in dry-season surface water versus flood-season counterparts, and markedly elevated concentrations in sediments relative to water column, underscoring medium-specific contamination gradients. Microplastic particles predominantly comprised transparent fibrous/fragmentary entities <500 μm, with polymeric constituents dominated by PP and PE. Urbanization-driven wastewater discharge emerged as the primary contamination vector. Notably, PLI assessment confirmed moderate pollution, whereas PHI and PERI analyses indicated elevated risks, with highly toxic polymers, such as PVC and PAN, contributing disproportionately to risk indices.

## 1. Introduction

Since 1950, plastics have been widely produced and used globally due to their inherent advantages, such as light weight, cost-effectiveness, and superior insulating properties [1], with production increasing exponentially [2]. This industrial proliferation, however, has triggered a critical environmental dilemma: post-consumer plastic waste degrades into microplastics that plastic fragments <5 mm [3] through mechanical abrasion, photochemical oxidation, and biological degradation [4] into the natural environment, causing serious environmental problems. Microplastics, owing to their small size, large surface area, and hydrophobicity, not only adsorb persistent organic pollutants and heavy metals [5], but are also easily ingested by organisms and transferred through the food chain, posing a potential threat to biological and human health.

In 2015, the United Nations included microplastics in its list of emerging pollutants, and their pollution status and ecological risks in the environment have become a focus of global research [6]. Rivers, serving as hydrological conduits for urban, industrial, and agricultural runoff, function as pivotal microplastic sinks and oceanic transport vectors. Approximately 80% of microplastic pollution in marine environments originates from terrestrial ecosystems closely associated with human activities [7]. Studies have reported that rivers such as the Mississippi, Amazon, Nile, Yangtze, Yellow River, and Pearl River all face severe microplastic pollution issues [8,9,10,11,12,13].

As the largest river in southeastern China, the Minjiang River serves as the primary recipient of industrial, agricultural, and domestic wastewater in its lower reaches. It also functions as a critical channel for transporting land-based pollutants from the southeastern coastal region to the ocean, playing a pivotal role in regional economic development and ecological security maintenance. In 2020, Huang et al. [14] investigated surface water from the mainstream to the estuary of the Minjiang River Basin to assess the influence of urbanization and dams on spatial variation in microplastics. Their study reported the highest average abundance of MPs in the Fuzhou urban section (1.97 ± 0.89 n/L), with dominant morphological characteristics being transparency, sizes of 1.0–2.0 mm, and fibrous shapes. PET and PE were the main polymer types. A positive correlation was also observed between microplastic abundance and the level of urbanization. However, only three widely distributed sampling points were established in the lower reaches of the Minjiang River, and microplastic pollution in sediment was not investigated. To more systematically and accurately evaluate microplastic pollution in the lower reaches of the Minjiang River, this study conducted a detailed investigation into the distribution characteristics of microplastics in surface water and sediments. Specifically, it analyzed the abundance, size, color, shape, and polymer composition, and for the first time, evaluated the ecological risks of microplastic pollution in the lower reaches of the Minjiang River. This study provides data support for establishing a sound microplastic management system for rivers and reducing microplastic environmental risks.

## 2. Materials and Methods

### 2.1. Study Area and Sample Collection

**Figure 1:**
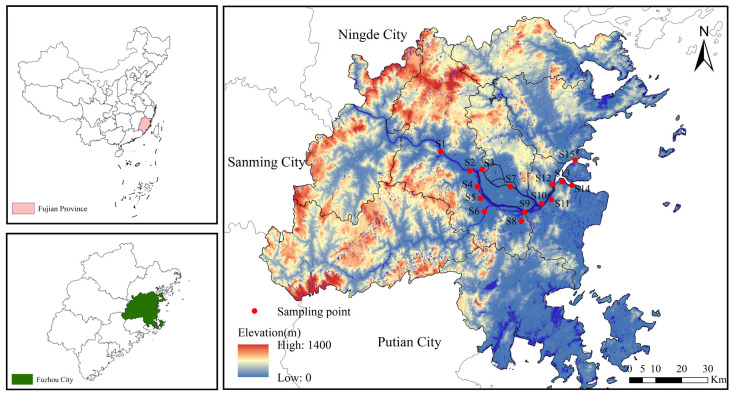
Overview of the study area and sampling point layout.

The Minjiang River originates form the Wuyi Mountains and empties into the East China. With a total basin area of 60,992 km^2^, approximately half the size of Fujian Province, and it provides water for 40% of the province’s economic output and drinking water for one-third of its population. The basin experiences a subtropical marine monsoon climate, featuring an average annual temperature of 16–20 °C and annual precipitation of up to 1617 mm. The lower Minjiang River starts at the Shuikou Power Station Dam in Shuikou Town and ends at the Minjiang River estuary, with a total length of approximately 113.7 km, all within the jurisdiction of Fuzhou City [14]. Guided by the hydrological characteristics and environmental backdrop of this river section, 15 sampling sites were set up at the confluence of the main stream and major tributaries (Figure 1). Surface water samples were collected during the flood season (July 2024) and dry season (November 2024), while sediment samples were collected in November of the same year.

Surface water samples (depth 0–30 cm) were collected using a 5 L stainless steel sampler with three replicate samples. After precisely measuring 15 L of surface water, the water was filtered through a 300-mesh stainless steel sieve. The retained material on the sieve was rinsed with ultrapure water, and the rinse solution was collected in a 500 mL brown glass bottle. The bottle should be sealed with aluminum foil, labeled properly, and transported to the laboratory immediately for analysis. Sediment samples were collected using a stainless-steel mud grabber. During the collection process, the mud grab was lowered to a depth of approximately 5–10 cm below the sediment-water interface. At each sampling site, three consecutive samples were collected, totaling 1 kg of sediment. Large stones, branches, and other debris were removed, and the sediment was sealed in aluminum foil bags. After recording the information, the samples were promptly transported to the laboratory for subsequent analysis.

### 2.2. Separation and Identification of Samples

Water sample processing: After thoroughly mixing 500 mL of the sample, transfer it to an 800 mL glass beaker. Add an adequate of saturated NaCl solution (1.20 g/cm^3^), bringing the liquid level close to the volume of the beaker. Thoroughly mix and perform density separation. After standing for 24 h, slowly pour out most of the solution in the beaker (about 600–700 mL), while the residual liquid containing sand grains and impurities at the bottom was discarded. Add 50 mL of 30% H_2_O_2_ solution to the transferred solution, seal with aluminum foil, and stand for 72 h to degrade organic matter [15]. Although H_2_O_2_ has a relatively small effect on most polymers at room temperature, in order to minimize its damage to microplastics as much as possible, this study adopted strict room-temperature and light-proof digestion conditions and avoided the use of metal catalysts or heating processes [16]. Following digestion, perform vacuum filtration through a 0.45-μm mixed cellulose filter membrane. Place the membrane in a 50 mm glass Petri dish, air-dry to constant weight, and label for subsequent microscopic analysis.

Sediment samples processing: Take 1 kg of sediment and dry it in a vacuum oven at 60 °C until constant weight is reached. Weigh 50 g of the dried sediment into a beaker, add 250 mL of saturated NaCl solution (1.20 g/cm^3^), and mix thoroughly for density separation. After 48 h of settling, vacuum filter the supernatant and rinse the residue back into the beaker [17]. Repeat the flotation procedure three times to maximize microplastic recovery from the sediment. Rinse the material on the membrane into a beaker, add 50 mL of 30% H_2_O_2_ solution, and digest for 72 h to degrade organic matter in the sediment. Subsequent processing follows the same as water samples.

Microplastics were counted and observed for their morphology, including shape, color, and particle size, using Leica stereo microscope (Leica DM 500) (Leica Microsystems Wetzlar GmbH, Vizla, Germany). The test filter membrane was placed on the stage, and scanned in a clockwise direction at 100× magnification across 50 non-overlapping fields of view, with images captured. Microplastics were identified according to specific standards [18]. Qualitative analysis of microplastics was performed using DXR3xi micro-Raman imaging spectrometer (Thermo Fisher Scientific, Inc., Waltham, MA, USA). Chemical composition analysis of suspected microplastics was conducted in single-point scanning mode, with polymer components identified by matching with the device’s built-in spectral database. The surface microstructure of microplastics was observed using Scanning Electron Microscope (German Zeiss Sigma 300 model) (Carl Zeiss AG, Oberkochen, Germany), and their surface elemental composition was determined through Energy Dispersive Spectroscopy (EDS). The EDS detection parameters maintained spatial consistency with the SEM observation conditions to ensure the correspondence between morphological features and elemental distribution.

### 2.3. Quality Control

To ensure the accuracy and reliability of the experimental data, strict quality control measures were implemented throughout the entire experimental process. Sample processing and identification were conducted in a clean laboratory and on a clean workbench. Experimental personnel wore cotton clothing during the operation. Two sets of parallel samples were set up at each sampling point. Samples were stored at 4 °C and analyzed and tested as soon as possible. Experimental tools and utensils were made of glass or stainless steel. They were rinsed at least three times with ultrapure water before use. Glassware used in the analysis process was covered with clean aluminum foil. Under the same experimental conditions, a blank sample was prepared by adding 500 mL of pre-filtered (0.45 μm) ultrapure water to a clean beaker and processed identically to the environmental samples. Analysis confirmed the absence of detectable microplastic particles in all blanks. Therefore, all microplastics identified in the environmental samples were considered to originate from the sampled media themselves, and no blank correction was applied to the reported abundance values.

### 2.4. Data Sources and Statistical Analysis

#### 2.4.1. Socioeconomic Data

GDP density (10,000 CNY/km^2^) data were acquired from the 2020 China GDP Spatial Distribution Kilometer Grid Dataset. Using ArcGIS 10.2 software, a 2 km buffer zone analysis was conducted around each sampling site to calculate the spatial average value. Population density (capita/km^2^) data were sourced from the 2022 Population Density Dataset jointly released by the National Qinghai–Tibet Plateau Science Data Centre and the Institute of Geographic Sciences and Natural Resources Research of the Chinese Academy of Sciences, with spatial matching achieved via the same buffer zone method. Nighttime light index data were obtained from the 2024 NOAA/VIIRS/DNB/MONTHLY_V1/VCMCFG dataset (500 m resolution) on the Google Earth Engine platform, with monthly average intensity values extracted from the 2 km buffer zone. Land use type data were based on the 2023 LUCC dataset from Wuhan University (30 m resolution), with the proportions of construction land, agricultural land, and forest land quantified through high precision buffer zone analysis. The results of the socioeconomic data analysis are shown in Table 1.

#### 2.4.2. Statistical Analysis

Nano Measure 1.2 software was employed for microplastic size statistics; IBM SPSS Statistics 21.0 was used for correlation analysis between microplastic abundance and other datasets; OMNIC 9.2 software was utilized for Raman spectroscopy analysis; relevant data plots were generated using Origin 2021; spatial analyses were conducted with ArcGIS 10.8. The abundance of microplastics was expressed as the number of microplastics per liter of water (n/L) and the number of microplastics per kilogram of sediment (n/kg).

### 2.5. Ecological Risk Assessment Methods

The ecological risk assessment of microplastics is a critical step in understanding their environmental impact and developing management strategies. Such assessments must not only examine their distribution and toxic effects within the environment but also comprehensively evaluate exposure pathways across different environmental media, their binding with harmful substances, their toxic effects on organisms, and their long-term impacts on ecosystems.

The Pollution Load Index (PLI) method integrates single-point pollution indices to calculate a regional composite index [19], which can reflect the pollution level of specific sampling points and comprehensively assess the overall regional pollution status, intuitively illustrating the contribution of each point to the regional pollution. The calculation formula are as follows:

(1) $${CF}_{i}=\frac{C_{i}}{C_{0}}$$

(2) $$PLI_{i}=\sqrt{{CF}_{i}}$$

(3) $$PLI_{y}=\sqrt[{n}]{PLI_{1}\times PLI_{2}\times \cdots \times PLI_{n}}$$

In these formulas, *CF_i_* denotes the microplastic pollution coefficient at the sampling point; *C_i_* represents the measured microplastic abundance at the sampling point (in n/L or n/kg); *C*_0_ is the background microplastic abundance (in this study, the minimum abundance of microplastics at each sampling point was adopted, in the same unit as *C_i_*); *PLI_i_* is the microplastic pollution load index at the sampling point, and *PLI_y_* is the microplastic pollution load index for the entire study area. Based on PLI values, microplastic pollution levels are categorized into 3 levels: <1 (mild pollution), 1–2 (moderate pollution), and >2 (severe pollution).

The Polymer Chemical Toxicity Hazard Index (PHI) combines the proportion of microplastic polymers in the environment and toxicity parameters, serving as a key tool for comprehensive risk assessment of microplastic pollution. The calculation formula is as follows:

(4) $$PHI=\sum P_{i}\times S_{i}$$

In this formula, *P_i_* is the percent of MP polymer types collected at each sampling station; *S_i_* denotes the hazard coefficient of the corresponding microplastic polymer. Researchers classified and assigned ratings to the potential ecological and health risks of 55 plastic polymers [20] based on the EU CLP Regulation (GHS system), as shown in Table 2. Based on PHI values, the ecological risk of microplastics is categorized into 5 levels: 0–1 (I), 1–10 (II), 10–100 (III), 100–1000 (IV), >1000 (V).

The Potential Ecological Risk Index (PERI) integrates the toxicity of a single pollutant with the combined effects of multiple components [21], establishing an assessment model and ecological risk classification based on microplastic abundance, polymer types, and ecological toxicity. The calculation formula are as follows:

(5) $${CF}_{i}=\frac{C_{i}}{C_{0}}$$

(6) $$T_{i}=\sum \frac{P_{n}}{C_{i}}\times S_{i}$$

(7) $$PERI=T_{i}\times {CF}_{i}$$

In these formulas, *CF_i_* represents the microplastic pollution coefficient at the sampling point; *C_i_* is the microplastic abundance at the sampling point; *C*_0_ is the background microplastic abundance; *T_i_* is the polymer toxicity coefficient; *P_n_* is the abundance of each polymer type; and S_i_ is the hazard coefficient of each polymer.

Based on PERI values, the ecological risk of microplastics is categorized into 5 levels: <150 (I), 150–300 (II), 300–600 (III), 600–1200 (IV), >1200 (V).

## 3. Results

### 3.1. Distribution Characteristics of MPs

#### 3.1.1. Abundance and Spatiotemporal Distribution of MPs

**Figure 2:**
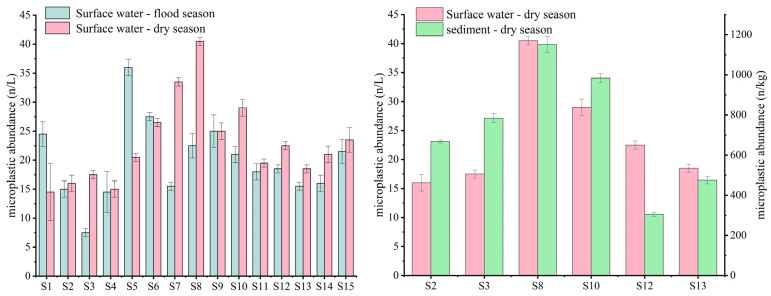
Abundance of MPs in surface water (n/L) and sediments (n/kg).

As shown in Figure 2, microplastics were detected at all sampling sites, with their abundance exhibiting significant spatiotemporal variation. The average microplastic abundance in surface water was 19.90 ± 1.56 n/L (flood season) and 22.87 ± 1.32 n/L (dry season), yielding an overall average of 21.38 ± 1.44 n/L for both seasons. Temporally, among the 15 sampling points, microplastic abundance was generally higher during the dry season than during the flood season. Notably, sites with higher abundance exhibited a greater inter-period difference, potentially attributed to reduced river discharge and flow velocity during the dry season, which weakens the water body’s dilution capacity for microplastics [22]. During the flood season, monsoons bring heavy rainfall, resulting in high river discharge, rapid flow velocity, and increased sediment content. Microplastics in aquatic environments readily migrate into sediments through sedimentation or undergo vertical diffusion driven by hydrodynamic forces. According to data from the Fuzhou Hydrological Station, the average annual runoff in the Min River basin is approximately 6.2 × 10^10^ m^3^. Runoff significantly decreases during the dry season, and its impact on the distribution of microplastic abundance at various points along the lower reaches of the Minjiang River cannot be overlooked. Spatially, microplastic abundance was higher in tributaries than in the main stream, while the estuary showed lower abundance with smaller inter-period variations. Tributaries have smaller water volumes, limited dilution and dispersion capacity, and are surrounded by dense pollution sources, leading to higher microplastic concentrations [23]. At the estuary, microplastic distribution is influenced by hydrodynamic and wetland ecosystems, exhibiting unique characteristics.

Due to the unique water environmental conditions of the urban rivers in the study area, sediment samples were unfeasible at certain sites, yielding a total of 6 sediment samples. Microplastic abundance in sediments ranged from 305.50 ± 10.61 to 1151.50 ± 40.31 n/kg, with an average abundance of 728.17 ± 20.51 n/kg, which is 30–50 times higher than that of surface water during the same period. The trends in microplastic abundance at different sampling sites generally aligned with those in surface water. Trend analysis indicated that, excluding the S13 Bat Island site, sediment microplastic distributions aligned with concurrent surface water trends, influenced by hydrological dynamics, pollution inputs, and ecosystem retention processes [24]. Compared to the dynamic migration environment of surface water, the low disturbance characteristics of sediments significantly reduce the risk of microplastic resuspension, providing a relatively stable enrichment environment for microplastics. Additionally, sediment sampling occurred during the low-flow season when reduced river velocities prolonged microplastic suspension time, further increasing their sedimentation probability.

#### 3.1.2. Levels of MP Pollution

Table 3 presents a comparison of microplastic pollution results between the Minjiang River and other global river systems. Compared with other typical freshwater environments, microplastic pollution in the lower Minjiang River remains at a moderately high level. The average microplastic abundance in surface water (21.38 ± 1.44 n/L) is comparable to that in China’s Taihu Lake (3.4–25.8 n/L), Poyang Lake (5–34 n/L), and Pakistan’s Ravi River (0.19–16.15 n/L), but notably lower than heavily polluted basins like the Baotou section of the Yellow River (432.5–2510.83 n/L) and India’s Mula River (1561 ± 167 n/L). Sediment microplastic abundance (728.17 ± 20.51 n/kg) aligns with urban rivers in Changsha, China (270.17 ± 48.23–867 ± 38 n/kg) and the UK’s Thames River (660 n/kg), yet is significantly lower than severely polluted systems such as the Yellow River’s Baotou reach (3766.67–6166.67 n/kg), the Netherlands’ Rhine River (60–10,500 n/kg), and the Mediterranean’s Ebro River (2052 ± 746 n/kg). The abundance of microplastics in global freshwater exhibits significant spatial heterogeneity, influenced by factors such as source loading, hydrodynamic conditions, and geographical factors.

#### 3.1.3. Morphological Characteristics of MPs

**Figure 3:**
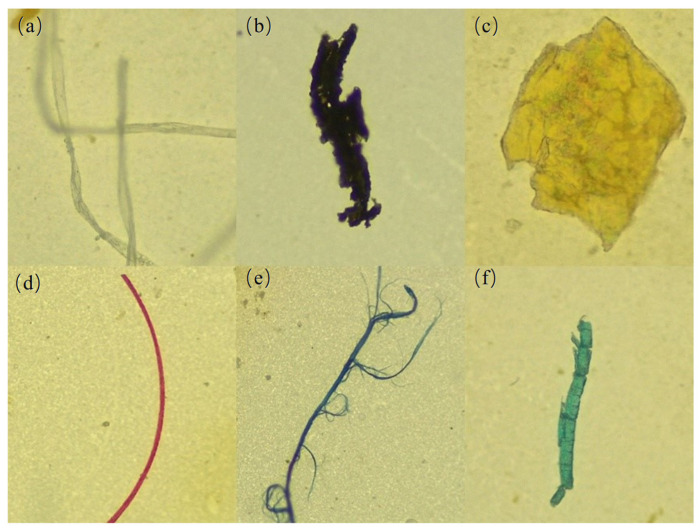
Morphological characteristics image of MPs: (**a**) transparent fibers, (**b**) black fragments, (**c**) yellow film, (**d**) red fibers, (**e**) blue fibers, (**f**) green fragments.

Based on morphological characteristics, microplastics are categorized into four shapes: fibers, fragments, films, and particles. In terms of color, they are classified into six groups: transparent, black, yellow, blue, red, and others. By size, microplastics are divided into four fractions: <500 μm, 500–1000 μm, 1000–3000 μm, and 3000–5000 μm. Based on filtration efficiency, microscope resolution and the need for reliable identification, microplastics smaller than 100 μm were excluded from statistical analysis in this study. As illustrated in Figure 3, microplastics shapes are primarily fibers and fragments, though notable differences exist between environmental media. In surface water, fibers are dominant (63.13% in the flood season and 48.79% in the dry season), whereas fragments dominate in sediments (51.51%). Fibers, with their slender morphology, experience reduced settling velocity due to hydrodynamic shear forces [53], allowing them to remain suspended in surface water. Conversely, fragment-shaped microplastics, often composed of high-density polymers, are more prone to gravitational setting and accumulation in sediments [54]. Film and granular microplastics are relatively scarce in both water and sediments of the lower Minjiang River. Fibrous microplastics have a relatively high specific surface area and are prone to entanglement and aggregation due to electrostatic effects. This not only makes them more likely to become carriers of pollutants but also increases the risk of accidental ingestion by organisms, thereby leading to physical blockages within the organisms. The edges of fragmented microplastics are usually sharp and have considerable potential for physical damage. Film-like microplastics are generally soft in texture and can be easily ingested by larger organisms. Moreover, due to their high content of additives, they have become an important source of chemical substance release [55].

Based on microplastic color distribution results, transparent microplastics were the most prevalent at all sampling sites, accounting for 47.77% (flood season surface water), 52.49% (dry season surface water), and 48.58% (dry season sediments), followed by black microplastics, with other colors being relatively rare. The high abundances of transparent and black microplastics correlate with the colors of commonly used plastic products and color fading due to plastic aging. Notably, the proportions of blue and red microplastics in sediments were significantly higher than in surface water, potentially because these pigments often contain surface-polar metal ions that enhance adsorption of organic matter and microorganisms. This leads to increased composite density [56], accelerating gravitational settling. In terms of size, microplastics predominantly measure < 500 μm, with surface water accounting for 90.25% (flood season) and 79.46% (dry season), and 79.28% in sediments. They exhibit a clear trend of increasing concentration with decreasing particle size. Microplastics in the environment undergo continuous fragmentation into smaller particles via multiple processes: UV oxidation from sunlight, hydrodynamic erosion, frictional wear from sediment interaction, and enzymatic degradation by microorganisms [57]. The proportion of microplastics > 1000 μm was lower in sediments than in surface water, possibly due to the transport dynamics of larger particles and bioturbation activities of benthic organisms.

**Figure 4:**
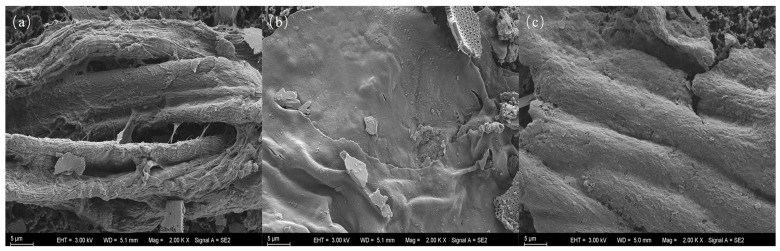
Representative SEM images of microplastics from surface water (**a**,**b**) and sediment (**c**) samples at 2000× magnification. (**a**) Fiber surface showing severe mechanical abrasion with visible deep grooves. (**b**) Fragment surface covered with dense cracks and a fractured network. (**c**) Film surface exhibiting adherent particles and micro-pores. All scales are 5 μm.

SEM observations revealed that microplastic surfaces exhibited curling, cracks, pores, and tears, evidence of substantial surface aging (Figure 4). These morphological features are likely the result of long-term ultraviolet radiation, sediment abrasion, and microbial activity [58]. The aging process not only alters the density and migration behavior of microplastics but also increases their specific surface area, thereby enhancing their adsorption capacity for coexisting pollutants such as heavy metals and persistent organic pollutants and potentially amplifying their ecological risks [59]. EDS analysis showed that the microplastic surface were primarily composed of carbon (C) and oxygen (O). Additionally, signals from metal elements such as aluminum (Al), potassium (K), titanium (Ti), iron (Fe), zinc (Zn), and mercury (Hg) were detected on some microplastics (Table 4), suggesting their potential adsorption from the external environment. Elemental composition data from EDS analysis are presented in Figures S1 and S2. Furthermore, microplastics with aging characteristics displayed a more diverse array of surface metallic elements. Aged microplastics feature increased specific surface area, elevated oxygen-containing functional groups, and enhanced surface electronegativity, collectively augmenting their adsorption capacity for metals.

#### 3.1.4. Polymer Composition and Distribution of MPs

**Figure 5:**
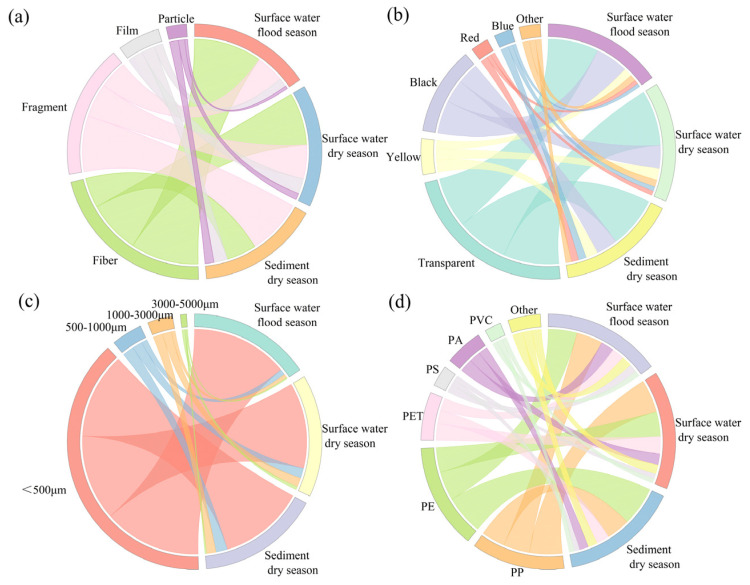
Morphological distribution of MPs: (**a**) shapes, (**b**) colors, (**c**) sizes, (**d**) polymer types.

In this study, a total of 570 suspected microplastics from surface water samples were selected for Raman spectroscopy detection, of which 449 were confirmed as microplastics, and twelve polymer types were identified in surface water: polypropylene (PP), polyethylene (PE), polyethylene terephthalate (PET), polystyrene (PS), polyamide (PA), polyvinyl chloride (PVC), polycarbonate (PC), polyvinyl alcohol (PVA), acrylonitrile-butadiene-styrene copolymer (ABS), ethylene-vinyl acetate copolymer (EVA), polyphenylene sulfide (PPS), and polytetrafluoroethylene (PTFE). As shown in Figure 5, PE and PP were the dominant polymers, showing relatively high proportions in both flood and dry seasons. Specifically, during the flood season, PE (27.60%) accounted for a higher proportion than PP (25.14%) in the flood season, while PP (31.32%) exceeded PE (23.77%) in the dry season. This alternation is attributed to their physical properties and migration behaviors under varying hydrological conditions. Other polymer types also displayed distinct seasonal variation patterns.

Meanwhile, 120 suspected microplastics were selected from sediment samples for identification, and a total of 91 microplastics were identified, including 9 types of polymers: PP, PE, PET, PS, PA, PVC, PC, EVA, and polyacrylonitrile (PAN). The dominant polymers were consistent with surface water, but PAN, which was not detected in the surface water, was identified in sediments. PAN has a density range of 1.18–1.22 g/cm^3^, significantly higher than freshwater, driving its gravitational migration toward sediments in aquatic environments. This density disparity facilitates PAN microplastics settlement in stagnant or low-flow conditions, reinforcing that microplastic density is a pivotal factor governing environmental migration.

### 3.2. Relationship Between MP Abundance and Human Activities and Water Quality Parameters

#### 3.2.1. Correlation Between MP Abundance and Socioeconomic Development Indicators

**Figure 6:**
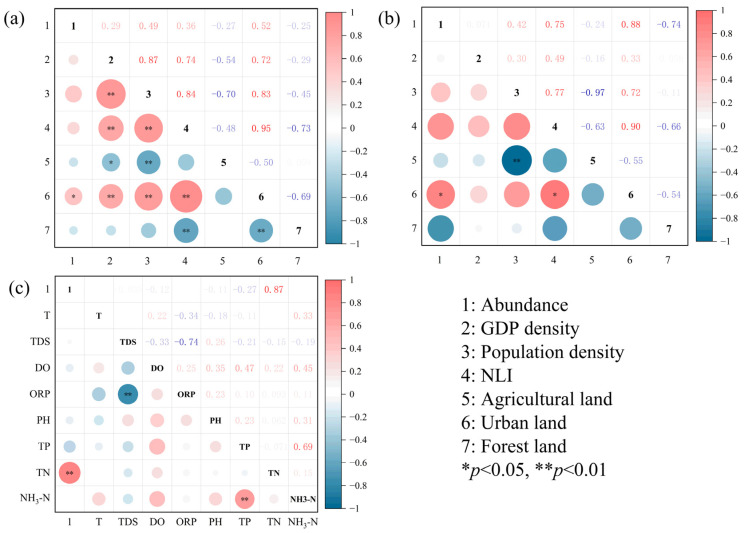
Correlation between MP abundance and human activity and water quality parameters (**a**) surface water—dry season, (**b**) sediments—dry season, (**c**) surface water—flood season.

The study findings show that during the dry season, microplastic abundance in surface water (r = 0.52, *p* < 0.05) and sediments (r = 0.88, *p* < 0.01) exhibits a significant positive correlation with the proportion of urban land use (Figure 6). Further analysis indicated that an increase in the proportion of urban land is accompanied by a rise in microplastic abundance in both surface water and sediments. From industrial production to daily life, urban areas serve as both the “production hub” for microplastics and the “distribution center” for their environmental dispersion. Surface water microplastics are highly susceptible to dynamic forces such as water currents and wind, exhibiting significant abundance fluctuations that reflect short-term pollution patterns. Conversely, microplastics gradually accumulate in sediments through sedimentation, reflecting historical pollution accumulation. The commonality between both forms and their correlation with urban land use proportions further indicates that human activities particularly urbanization are the underlying drivers of microplastic pollution.

It is worth noting that although GDP density and population density are positively correlated with microplastic abundance (*p* > 0.05), they do not reach a statistically significant level. We speculate that this phenomenon might be attributed to several factors. Firstly, the spatial heterogeneity of pollution sources is relatively low. Under the constraints of the basin’s hydrodynamic conditions, the input sources of microplastics along the riverbanks have strong common sources, which weakens the influence of local economic and population differences on their spatial distribution. Secondly, after entering water bodies, microplastics are prone to sedimentation or longitudinal transportation due to water flows. As the spatial range expands, the direct connection between the abundance of microplastics and GDP density as well as population density tends to weaken. Finally, there may be a threshold effect in socio-economic driving factors; When the urbanization level of a certain area reaches a certain extent, the change in the abundance of microplastics may rely more on the types of urban activities, such as industrial structure and consumption patterns, rather than a simple increase in economic aggregate or population density.

#### 3.2.2. Correlation Between MP Abundance and Water Quality Parameters

As shown in Figure 6c, during the flood season, during the flood season, microplastic abundance in surface water showed a significant positive correlation with TN (r = 0.87, *p* < 0.01), but no significant association with TP or NH_3_-N. Nitrogen sources in rivers primarily originate from agricultural activities, wastewater treatment plant discharges, and industrial production, further reinforcing the close link between microplastic pollution and human activities. No significant correlations were observed between other water quality parameters and microplastic abundance. This may stem from the high complexity of river ecosystems, coupled with substantial fluctuations in flow velocity within the study area. Variations in water flow speed can significantly influence microplastic migration pathways and distribution patterns in rivers, thereby affecting correlations with water quality parameters. Alternatively, the small sample size of this study may have prevented the accurate detection of weak associations between microplastics and water quality parameters.

### 3.3. Ecological Risk Assessment of MPs

**Figure 7:**
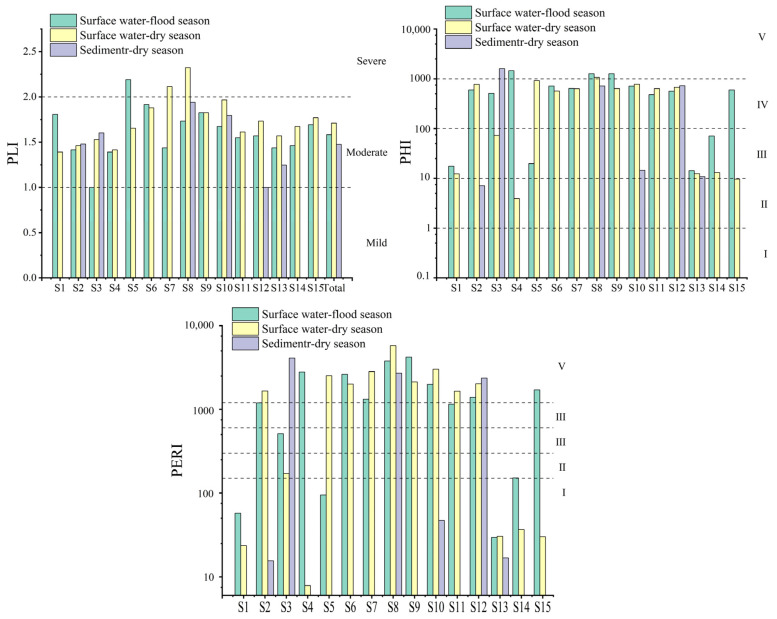
PLI, PHI, and PERI of MPs in surface water and sediments.

As shown in Figure 7, the study area exhibited average PLI values of 1.58 (flood season surface water), 1.71 (dry season surface water), and 1.48 (dry season sediments), all indicating moderate pollution. Pollution intensity was higher in the dry season than in the flood season, with surface water generally showing higher microplastic pollution load indices than sediments. The PLI’s spatial distribution mirrored microplastic abundance, indicating that the index effectively reflects pollution load. However, its failure to account for toxicity disparities may lead to underestimation of risks at highly toxic sites. For instance, S12 was categorized as moderately polluted by PLI, but its PERI reached Grade V. PHI focuses on the toxicity contributions of different polymer types. The study area’s ecological risk stood at the relatively high Grade IV, primarily driven by highly toxic polymers such as PVC (10,001), PAN (11,521), PA (47), and PPS (897). Peak risk levels varied across media and seasons, reflecting the influence of seasonal pollution source inputs on microplastic ecological risk. Notably, this method may produce fluctuating results due to the presence of trace amounts of highly toxic polymers.

The PERI integrates microplastic abundance, environmental thresholds, and toxicity coefficients to conduct comprehensive ecological risk assessments. Results show average PERI values of 1535.81 (flood season surface water), 1592.99 (dry season surface water), and 1542.80 (dry-season sediments), all reaching Grade V (extremely high risk). The risk hierarchy is as follows: dry-season surface water > dry-season sediments > flood-season surface water. The highest PERI in dry-season surface water stems from elevated microplastic abundance and intensified toxic substance inputs. Sediments rank second due to long-term accumulation and pollutant adsorption, while the flood season shows the lowest risk, attributed to dilution, enhanced migration, and seasonal variations in pollution sources. Notably, S8 and S12 exhibit particularly acute ecological risks. Despite stark differences in microplastic abundance, both sites harbor highly toxic polymers. S12 specifically contains PAN components with extremely high hazard coefficients, indicating that toxicity coefficients are the decisive factor in ecological risk, rather than abundance alone.

## 4. Discussion

### 4.1. Analysis of Potential Sources of MPs

The composition of microplastics in different freshwater and sediment samples exhibits remarkable variation, which is closely linked to local determinants such as urbanization level, economic diversity, and population density. The lower Minjiang River traverse rural, industrial, and urban zones, with microplastic sources encompassing domestic wastewater discharge, agricultural practices, tire abrasion from shipping, and atmospheric deposition. Among these, synthetic textiles like polyester and acrylic serve as the primary sources of fibers in laundry wastewater. Studies indicate that washing 5 kg of polyester clothing can release up to 6 million microfibers [60]. Raman spectroscopy analysis revealed that surface water fibers were predominantly polyamide (PA), consistent with fibers released from laundry wastewater. Huang et al. [61] also confirmed in their study of the Yangtze River basin that clothing washing is the primary source of fibrous microplastics. Fragment-type microplastics derive from the decomposition of large plastic waste, primarily as small colored fragments from the improper disposal of common packaging bags and plastic containers in daily life.

The sources and compositions of microplastics differ among sampling sites. S6 and S8 tributaries of the lower Minjiang River, are surrounded by extensive farmland. Field investigations showed that abundant discarded agricultural films piled along farmland edges, with their main polymer components being polyethylene (PE) and its copolymers [62]. studied microplastics in farmland soils in Hangzhou Bay and found that microplastic concentrations in covered soils were higher than in uncovered soils, and the proportion of films in covered soils was significantly higher. Additionally, the dry season is the peak period for plastic film application in vegetable greenhouses [63], and farmland runoff carrying plastic film fragments significantly increased microplastic abundance at the S8, reflecting seasonal variations in agricultural non-point source pollution. Microplastics at the S7 mainly originated from urban and tourist waste, such as plastic food containers, beverage bottles, and packaging bags; At the S12, the detection of PAN is likely associated with the degradation of local agricultural insect nets, reflecting the pollution source specificity of different functional zones. In the Minjiang River estuary (S11–S15), microplastics primarily originate from tidal dynamics and marine input. Tidal dynamics transports plastic debris such as fishing buoys and shipping waste into the estuary [54], where these materials degrade into microplastics under ultraviolet radiation and hydrodynamic forces. Tidal also mixes seawater with river water, leading to more homogeneous microplastic distribution. Additionally, the Minjiang River estuary wetland is the northern boundary of mangrove distribution along the Asian continental coast., while microplastics settle due to gravitational forces and are intercepted by mangrove root systems as flow velocity decreases [64], discarded fishing nets and plastic buoys from surrounding aquaculture activities still infiltrate the waterbody via drainage systems.

### 4.2. Pollution Risks of MPs and Prevention and Control Measures

Microplastics contain inherent chemical additives like colorants and plasticizers. Due to their hydrophobic properties and large specific surface area [65], they can adsorb other chemicals, such as heavy metals and persistent organic pollutants, forming composite pollutants. These substances are difficult to decompose in the environment, prone to bioaccumulation in living organisms, and can enter the human body through the food chain. Studies have confirmed the detection of microplastics in human lungs [66], blood [67], and placentas [68]. This study also identified trace amounts of highly toxic PVC and PAN components in samples. PVC, one of the most widely used polymers, is commonly applied in watercraft, aquaculture equipment, and fishing vessel components operating in rivers and coastal areas. Pure PVC is hard and unstable, and a large number of additives must be added to form it [69]. Among them, plasticizers and heat stabilizers are of concern. Therefore, the quality risk of PVC itself may be underestimated. Its true ecological risk stems from the continuous release of toxic additives as a carrier. PAN, renowned for its UV resistance, is mainly used for synthetic fiber production. Both PVC and PAN are environmentally persistent, and importantly the International Agency for Research on Cancer (IARC) of the World Health Organization has classified PVC as a Group 3 carcinogen. The presence of trace toxic polymers in the environment highlights the urgent need to consider the environmental impacts of plastic materials and products after their use.

The Minjiang River is the largest river flowing into the sea in Fujian Province, China, with its lower reaches spanning the entire city of Fuzhou, serving as a core link for regional sustainable development. It plays a crucial role in residential water supply, agricultural irrigation, industrial production, and water transportation. For the prevention and control of microplastic pollution in the lower Minjinag River, key source-oriented measures include advanced treatment of industrial wastewater and domestic sewage, establishing a recovery mechanism for agricultural plastic mulch, and intercepting urban non-point source pollution like installing gratings in stormwater drainage systems. Special emphasis should be placed on promoting clean production in industries such as textiles and plastic processing within the basin, and replacing single-use plastic products with biodegradable materials. Additionally, restoring riverbank vegetation buffer zones and wetland systems can utilize microplastic concentrations in water bodies through ecological adsorption; The establishment of cross-regional joint prevention and control mechanisms, by leveraging automatic water quality monitoring stations and pollution dispersion models, can enhance risk warning capabilities. The implementation of microplastic pollution prevention and control requires collaborative efforts among policy and legal frameworks, technological innovation, and public participation to achieve the goal of systematic governance from pollution control to ecological restoration.

### 4.3. Density Separation Methods and Recycling of MPs

In this study, saturated NaCl solution (1.20 g/cm^3^) was used for density separation. This method is mainly applicable to the recovery of microplastics with densities lower than this value, such as PE (0.92–0.97 g/cm^3^) and PP (0.90–0.91 g/cm^3^). However, in the sample analysis, we detected polymers with a density higher than 1.20 g/cm^3^, such as PVC (1.16–1.58 g/cm^3^) [70]. This phenomenon may be related to the widespread aging of microplastics in the natural environment: After being subjected to weathering and abrasion, microplastics tend to develop pores and cracks on their surfaces, significantly reducing their apparent density and enabling some originally high-density particles to remain buoyant [58]. Although PVC contains substantial amounts of plasticizers, long-term leaching can generate internal pores, potentially facilitating microplastics into smaller, irregularly shaped particles and altering their settling behavior. Moreover, most microplastics observed in this experiment were fibers and fragments. For slender fibers in particular, the average settling velocity is considerably lower than that of equivalent spherical particles [71], which may explain the recovery of high-density fibrous PET or PVC particles along with the solution within the limited settling time. Therefore, the detection of high-density polymers such as PVC in this study, on the one hand, reflects the limitations of current methods in the recycling efficiency of high-density microplastics, and on the other hand, provides indirect evidence for the widespread aging of microplastics in the study area. In subsequent studies, we will adopt separation liquids with higher density, such as NaI (1.6–1.8 g/cm^3^) or ZnCl_2_ (1.5–1.7 g/cm^3^) solutions, to capture different types of polymers more comprehensively.

## 5. Conclusions

The spatiotemporal distribution of microplastics across sampling sites reveals that microplastic pollution in the lower Minjiang River ranks at a moderately high level globally. Surface water microplastics abundance exhibits the characteristics of being higher during the dry season than the flood season and higher in tributaries than in the main river. Concurrently, sediment microplastic concentrations significantly exceed those in surface water. Microplastics predominantly occur as transparent fibers or fragments with particle sizes < 500 μm, with polypropylene (PP) and polyethylene (PE) as the dominant polymer components. The significant correlation between microplastic abundance and urban land use (*p* < 0.05) confirms that anthropogenic activities, rather than natural factors, drive pollution in this region. Notably, flood-season microplastic abundance showed a strong positive correlation with TN (*p* < 0.01), indicating shared sources with nitrogenous wastewater and potential linkage to water eutrophication. Three risk assessment methods revealed that microplastic ecological risks are driven by both “accumulation” and “toxicity effects,” with polymer toxicity being decisive. The PLI indicates moderate risk, whereas the PHI and PERI both signaled higher risk levels. Even in areas with moderate microplastic abundance, trace amounts of highly toxic polymers may significantly elevate ecological risk, and traditional assessments based solely on abundance may underestimate actual hazards.

So far, only surface water and sediment samples have been collected in this study. Future research can further carry out vertical stratification of water bodies and sediment profile sampling to reveal the distribution patterns of microplastics in water bodies and sediments at different depths. Meanwhile, the detection of microplastics in organisms such as fish and benthic organisms can be combined to explore the migration and accumulation of microplastics in the three media of water—sediment—organisms. In addition, this study conducted a preliminary detection of the metal elements adhering to the surface of microplastics. As a semi-quantitative elemental analysis technique, EDS has a detection limit of approximately 0.1% to 1.0% for most metal elements. However, it is difficult to distinguish whether metals exist as additives inside microplastics or as adsorbents or attachments on the surface of microplastics. Subsequently, more precise analysis techniques such as XPS or ICP-MS can be combined to further confirm the chemical form and source of the metal. In terms of risk assessment, this study mainly evaluates based on the current environmental concentration. However, from a dynamic perspective, microplastics, as persistent pollution sources in rivers, even if their current concentrations are not high, their long-term accumulation and slow-release effects may still pose chronic toxicity risks. Therefore, chronic exposure experiments can be carried out in the future to more truly reflect the risks of microplastics in the environment.

## Figures and Tables

**Table 1:** Socio-economic development indicators for each sampling site.

Points	GDP Density (10,000 Yuan/km^2^)	Population Density (Capita/km^2^)	NLI (nw/cm^2^/sr)	Proportion of Land Use Types (%)		
				Agricultural Land	Urban Land	Forest Land
S1	3513	107	1.55	23.37	4.88	46.01
S2	4029	990	10.53	36.74	25.98	5.99
S3	107,124	1576	16.33	33.98	37.77	17.91
S4	17,698	2452	25.51	25.80	44.06	0.47
S5	9080	584	22.78	39.04	37.44	5.75
S6	4146	1004	14.56	51.35	29.67	1.05
S7	200,082	8759	44.63	5.13	79.25	0.00
S8	3583	1560	12.58	32.88	43.27	15.60
S9	19,494	2553	10.56	17.73	24.20	11.54
S10	43,649	3068	21.66	6.73	44.54	6.56
S11	16,306	845	13.30	25.11	37.92	27.46
S12	24,248	1854	5.33	21.43	19.88	57.55
S13	15,059	394	3.47	44.69	8.51	27.07
S14	13,978	288	3.05	34.41	7.67	52.95
S15	6046	546	4.50	36.55	15.78	39.70

**Table 2:** Hazardous factors of common polymers.

Name	Abbreviations	Hazard Factor	Risk Category
Polyethylene	PE	11	Medium
Polypropylene	PP	1	Negligible
Polyethylene glycol terephthalate	PET	4	Low
Polyamide	PA	47	Medium
Polyvinyl chloride	PVC	10,001	Very High
Polystyrene	PS	30	Medium
Ethylene vinyl acetate	EVA	9	Low
Polycarbonate	PC	610	High
Acrylonitrile butadiene styrene	ABS	6552	Very High
Polyacrylonitrile	PAN	11,521	Very High
Polyphenylene sulfide	PPS	897	High

**Table 3:** Abundance of MPs in surface freshwater and sediments in selected regions.

	Research Area	Region	Abundance	References
Surface Water	Mississippi River	USA	14–83 n/L	[11]
	Nile River	Egypt	0.5–4.38 n/L	[12]
	Amsterdam Canal	The Netherlands	0.67–11.53 n/L	[25]
	Thames River	UK	0.33–12.27 n/L	[26]
	Mula River	India	1561 ± 167 n/L	[27]
	Buriganga River	Bangladesh	4.33–43.67 n/L	[28]
	Ravi River	Pakistan	0.19–16.15 n/L	[29]
	Nakdong River	Republic of Korea	0.293–4.760 n/L	[30]
	Ottawa River	Canada	0.05–0.24 n/L	[31]
	Ciwalengke River	Indonesia	5.85 ± 3.28 n/L	[32]
	Pearl River	China	0.38 ± 7.92 n/L	[33]
	Northwest Wei River	China	3.67–10.7 n/L	[34]
	Yangtze River Estuary	China	1.71 ± 3.12 n/L	[35]
	Haihe River	China	2.64–18.45 n/L	[36]
	Taihu Lake	China	3.4–25.8 n/L	[37]
	Poyang Lake	China	5–34 n/L	[38]
	Three Gorges Reservoir	China	1.597–12.611 n/L	[39]
	Yellow River Baotou Section	China	432.5–2510.83 n/L	[40]
	**Minjiang River**	**China**	**21.38 ± 1.44** n/L	**This study**
Sediment	Nakdong River	Republic of Korea	1970 ± 62 n/kg	[30]
	Thames River	UK	660 n/kg	[41]
	London City Lakes	UK	539 n/kg	[42]
	Dutch River	The Netherlands	68–10500 n/kg	[43]
	Main River	Germany	786–1368 n/kg	[44]
	Ebro River	Mediterranean	2052 ± 746 n/kg	[45]
	Ganga River	India	99.27–409.86 n/kg	[46]
	Ontario Lake	Canada	4635 n/kg	[47]
	Ottawa River	Canada	220 n/kg	[31]
	Chiusi Lake	Italy	234 ± 85 n/kg	[48]
	Zahuapan River	Mexico	1633.34 ± 202.56 n/kg	[49]
	Pearl River	China	80–9597 n/kg	[33]
	Northwest Wei River	China	360–1320 n/kg	[34]
	Qinghai–Tibet Plateau’s River	China	50–195 n/kg	[50]
	Changsha City Rivers	China	270.17 ± 48.23–866.59 ± 37.96 n/kg	[51]
	Yangtze River Estuary	China	20–340 n/kg	[21]
	Taihu Lake	China	11.0–234.6 n/kg	[37]
	Qinghai Lake	China	50–1292 n/kg	[52]
	Poyang Lake	China	54–506 n/kg	[38]
	Three Gorges Reservoir	China	25–300 n/kg	[39]
	Yellow River Baotou Section	China	3766.67–6166.67 n/kg	[40]
	**Minjiang River**	**China**	**728.17 ± 20.51** n/kg	**This study**

**Table 4:** Surface elemental composition (Atomic %) of individual fibrous microplastics from surface water samples as determined by EDS analysis.

Elements	C	O	F	Al	Si	K	Ca	Ti	Fe	Zn	Hg
At (%)	54.61	40.19	0.37	0.60	2.38	0.14	0.05	0.02	0.40	0.30	0.95

## Data Availability

The datasets used and analyzed during the current study are available from the corresponding author on reasonable request.

## Supplementary Materials

The following supporting information can be downloaded at: https://www.mdpi.com/article/10.3390/toxics13121033/s1, Figure S1: Electron image and corresponding EDS spectrum of fibrous microplastics from surface water. Scale bar = 25 μm. The four elements with the highest atomic percentages detected on the fiber surface are listed on the right; Figure S2: Electron image and corresponding EDS spectrum of film-shaped microplastics from sediment. Scale bar = 25 μm. The four elements with the highest atomic percentages detected on the film surface are listed on the right.
