# Supplementary material for: Distribution, Sources, and Ecological Risk Assessment of Microplastics in the Lower Minjiang River
Source: Toxics. 2025 Nov 29;13(12):1033. doi: 10.3390/toxics13121033 (PMC12737208; doi:10.3390/toxics13121033)
Supplement: Supplementary file 1 [file toxics-13-01033-s001.zip › toxics-3960525-supplementary.pdf]

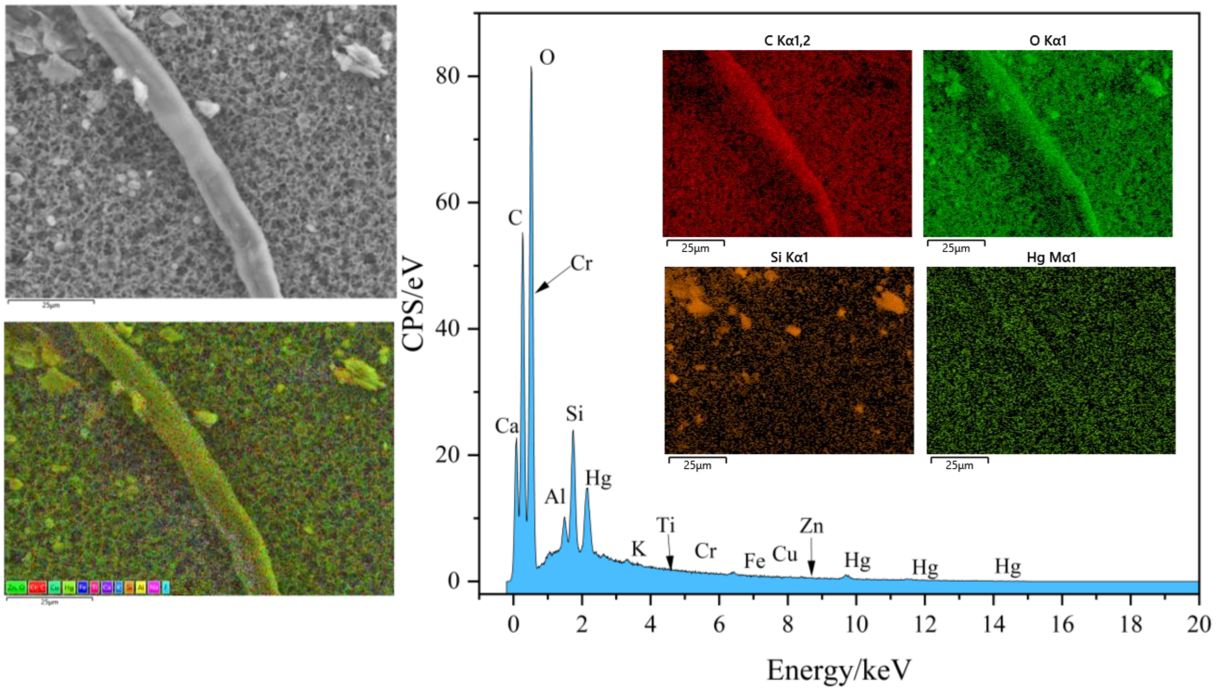

Figure S1. Electron image and corresponding EDS spectrum of fibrous microplastics from surface water. Scale bar = 25 μm. The four elements with the highest atomic percentages detected on the fiber surface are listed on the right.

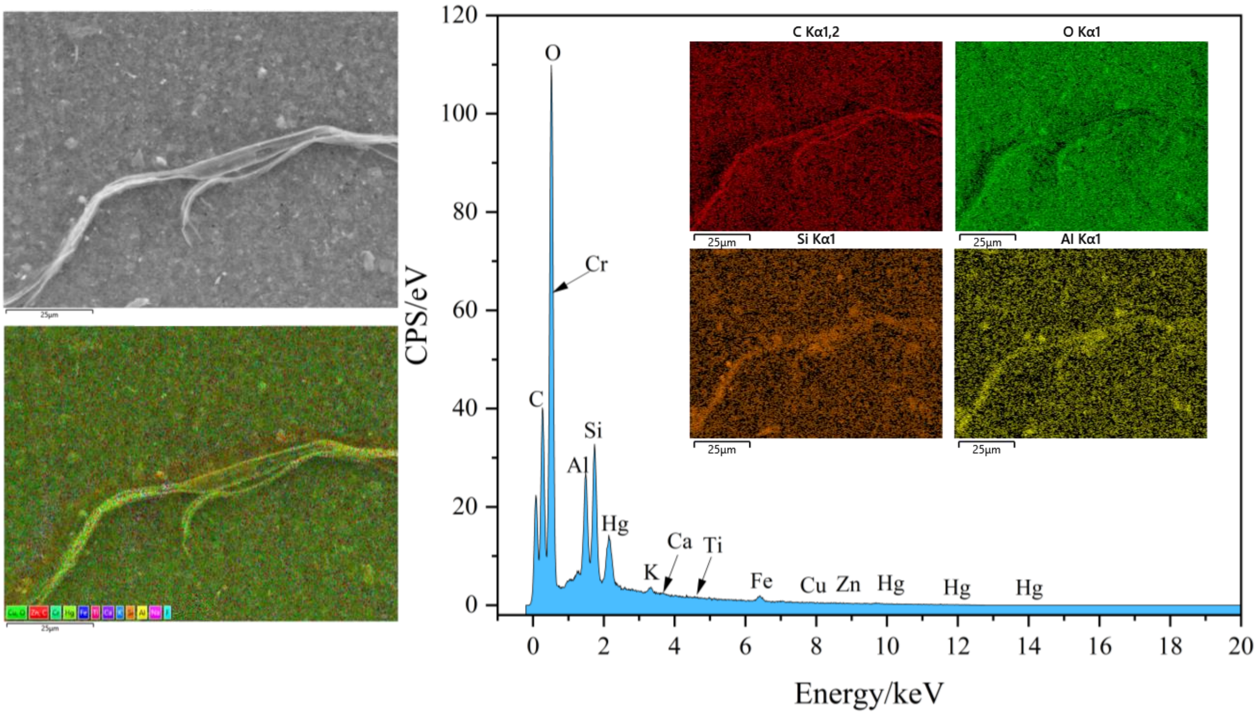

Figure S2. Electron image and corresponding EDS spectrum of film-shaped microplastics from sediment. Scale bar = 25 μm. The four elements with the highest atomic percentages detected on the film surface are listed on the right.
